# Kinetics of Serum-Free Light Chain Removal by High-Cutoff Hemodialysis in Patients with Multiple Myeloma and Acute Renal Failure

**DOI:** 10.3390/medicina61111977

**Published:** 2025-11-04

**Authors:** Wilma A. Veldman, Debora J. Weerman, Saskia Molog, Adry Diepenbroek, Wilfried W. H. Roeloffzen, Coen A. Stegeman, Casper F. M. Franssen

**Affiliations:** 1 Department of Internal Medicine, University Medical Center Groningen, University of Groningen, Groningen 9713 GZ, The Netherlands; w.veldman@umcg.nl (W.A.V.); d.j.weerman@umcg.nl (D.J.W.); s.molog@umcg.nl (S.M.); a.diepenbroek@umcg.nl (A.D.); c.a.stegeman@umcg.nl (C.A.S.); 2 Department of Hematology, University Medical Center Groningen, University of Groningen, Groningen 9713 GZ, The Netherlands; w.w.h.roeloffzen@umcg.nl

**Keywords:** cast nephropathy, hemodialysis, high cut-off membrane, multiple myeloma

## Abstract

*Background and objectives*: Cast nephropathy is the main cause of acute renal failure in patients with multiple myeloma. There are conflicting data on whether removal of serum free light chains (sFLCs) with a high-cutoff (HCO) dialyzer has a favorable effect on the recovery of renal function. This may in part be explained by differences in the efficacy of sFLC removal by HCO dialysis and treatment responses to anti-plasma cell therapy between studies. We studied the removal of sFLCs during HCO treatment in detail in relation to treatment response. *Materials and methods*: Pre-dialysis serum and dialysate levels of sFLCs were simultaneously and repeatedly measured during the first two HCO treatments in 10 patients with kappa (κ)- and 5 patients with lambda (λ)-producing myeloma that presented with dialysis-dependent renal failure at our institution between 2009 and 2024. *Results*: The average change in sFLCs during 6 h treatments was −57 ± 13%, but it varied widely between −29% and −77%. Mean reductions in sFLCs were comparable for κ and λ (−61.4 ± 19.1% and −55 ± 16.7%, respectively; *p* = 0.78). The average clearance of sFLCs at 15 min after the start of HCO dialysis was 42.1 ± 8.5 and 27.4 ± 15.6 mL/min for κ and λ, respectively (*p* < 0.01). Clearances decreased to 27.2 ± 11.3 for κ and 13.8 ± 7.9 mL/min for λ after 6 h of HCO treatment (*p* = 0.042). Renal function recovered in 11 patients (73%). In three of the four patients whose renal function did not recover, sFLC levels were >5 g/L at any time beyond 2 weeks after the start of HCO treatment. *Conclusions*: Although the clearance of κ was higher compared to λ, reductions in sFLCs were similar for κ and λ. We speculate that this discrepancy is explained by greater adherence of λ to the HCO membrane. Patients whose renal function did not recover had less of a reduction in sFLC levels during HCO treatment, probably due to a suboptimal hematological response to anti-plasma cell therapy.

## 1. Introduction

Light chain cast nephropathy is the main cause of dialysis-dependent acute renal failure in patients with multiple myeloma, accounting for approximately 90% of cases [1,2,3]. Cast nephropathy is caused by the precipitation of filtered serum-free light chains (sFLCs) in kidney tubules, resulting in cast formation, obstruction and peritubular inflammation [1,2,4]. Acute renal failure is a major risk factor for mortality in this patient group, and subsequent recovery of kidney function is associated with a better prognosis [1,2,5]. The aim of the initial treatment of patients with myeloma cast nephropathy is a rapid decrease in sFLCs, since an early reduction in sFLCs is associated with recovery of renal function [1,2,6]. Treatment consists of anti-plasma cell therapy to stop the overproduction of sFLCs and facilitating renal clearance of sFLCs through optimal hydration, treatment of hypercalcemia (if present) and avoiding nephrotoxic medication [1]. 

sFLCs can be effectively removed by a dialyzer with a high-cut-off (HCO) membrane [1,2,4,5,6,7,8,9,10,11,12]. Initial observational studies with HCO treatment showed promising results regarding renal recovery to the point that dialysis could be stopped [3,9]. However, subsequent randomized controlled studies comparing hemodialysis using HCO dialyzers with standard dialyzers showed conflicting results regarding renal recovery [4,12]. This may in part be explained by differences in the efficacy of sFLC removal during HCO treatment and differences in treatment response to anti-plasma cell therapy between studies. For a better understanding of the factors that are associated with the removal of sFLCs, we studied the course of kappa (κ) and lambda (λ) sFLCs during HCO treatment in detail, as well as the clearances by HCO treatment in relation to hematological treatment response. We also studied whether the clearance of sFLCs decreases during treatment because sFLCs may adhere to the membrane and clog the pores of the semipermeable membrane.

## 2. Materials and Methods

### 2.1. Study Design and Participants

In this observational study, all patients with acute renal failure due to cast nephropathy that were treated with hemodialysis at our institution between 2009 and 2024 were included. In this period, each patient with cast nephropathy that had an indication for hemodialysis was treated with an HCO dialyzer as part of routine patient care. This study was performed according to the principles of the Declaration of Helsinki. 

### 2.2. Chemotherapy

In all patients, anti-plasma cell therapy was initiated before HCO treatment was started since removal of sFLCs by HCO hemodialysis is only meaningful if the overproduction of sFLCs is inhibited. Standard anti-plasma cell therapy was a combination of bortezomib and dexamethasone in all patients. Three patients received additional anti-plasma cell treatment (lenalidomide in one patient and daratumumab in two patients), as detailed in Table 1. 

### 2.3. HCO Treatment

Patients were treated with the Theralite 2100 dialyzer with a surface area of 2.1 m^2^ (Baxter, Brooklyn Park, MN, USA). The duration of each treatment was 6 h. The dialysate composition was adjusted in response to the results of the plasma potassium and calcium concentrations. The dialysate potassium concentration varied between 1 and 3 mmol/L; dialysate calcium was 1.5 or 1.25 mmol/L. The standard dialysate concentration of sodium was 139 mmol/L. We used a relatively low dialysate concentration of bicarbonate of 30 mmol/L to avoid metabolic alkalosis during and after the 6 h hemodialysis treatment. Standard anticoagulation was unfractionated heparin using a bolus of 500 or 1000 units at the start of treatment and, next, a continuous infusion of 500 or 750 IE/h. In patients with increased bleeding risk, only a heparin bolus of 500 or 1000 IE was used. Hemodiafiltration was not used, since this would likely result in a more albumin loss through the HCO membrane. Dry weight was assessed clinically; ultrafiltration volume was set to achieve dry weight at the end of the treatment with a maximum ultrafiltration volume of 4 L during the 6 h treatment. In contrast to the studies by Hutchinson [4] and Bridoux [12], we did not routinely administer albumin during or after HCO hemodialysis.

Hemodialysis with HCO was started daily and tapered to every other day when sFLC were 1000–2000 mg/L. HCO treatment was stopped when the concentration of sFLC before the start of HCO treatment was <1000 mg/L during two consecutive days. Patients who were still dialysis-dependent despite sFLC concentrations <1000 mg/L before the start of 2 consecutive HCO treatments were transferred to hemodialysis with a standard dialyzer. 

### 2.4. Measurement of sFLCs and Their Clearance by HCO Dialysis

sFLCs were measured by nephelometry using the Freelite immunoassay (The Binding Site, Birmingham, UK). Creatinine clearance was calculated using serum creatinine and creatinine excretion in 24 h urine collections.

Pre-filter and post-filter sFLC concentrations and dialysate levels of FLCs were simultaneously and repeatedly measured during the first two HCO treatments: at the start of treatment and after 15, 60, 120, 240 and 360 min. During subsequent HCO treatments, only pre- and post-treatment plasma concentrations of sFLC were measured. 

Clearances of sFLCs during HCO treatment were calculated as follows: Clearance = Q_do_ × (C_do_/C_bi_). In this equation, Q_do_ denotes the efferent dialysate flow, which is a composite of afferent dialysate flow (500 mL/min in all treatments) and the ultrafiltration rate; C_do_ and C_bi_ denote the sFLC concentration in the efferent dialysate and the afferent blood, respectively. Notably, this clearance formula is based on the amount of sFLCs that diffuses to the dialysate per unit time and does not capture removal of sFLCs that could potentially adhere to the HCO membrane.

### 2.5. Statistical Analysis

Data were analyzed with Prism version 10 (GraphPad, San Diego, CA, USA). Data are expressed as means (±SDs) or medians (interquartile ranges (IQRs)). Comparisons between patients with κ- and λ-producing myeloma were made using the Mann–Whitney U test. Comparisons of plasma albumin concentrations before and after 7 days of HCO treatment were made with a paired *t*-test. *p* < 0.05 was considered statistically significant.

## 3. Results

### 3.1. Patient Characteristics

A total of 15 patients were included in this study. The diagnosis of cast nephropathy was made clinically. The mean (SD) age was 67.5 ± 9.6 years, and 12 patients were male. Ten patients had κ sFLCs and five had λ sFLCs (Table 1). Ten patients were newly diagnosed, and five patients had a relapse of a previously diagnosed multiple myeloma (Table 1). The mean interval between diagnosis and start of HCO treatment was 4.4 ± 4.8 (range 1 to 15) days for newly diagnosed patients and 1.4 ± 0.5 (range 1 to 2) days for patients with a relapse. In all patients, anti-plasma cell therapy was initiated before the first HCO treatment. The average number of HCO treatments per patient was 11 (range 4 to 39) (Table 1). 

### 3.2. Effect of a Single HCO Dialysis on SFLCs

**Figure 1:**
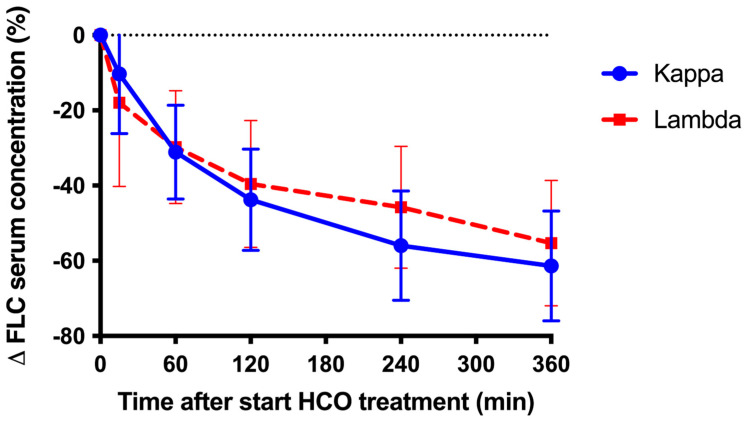
Relative change in sFLC during the first HCO treatment for kappa (10 patients) and lambda (5 patients). Error bars indicate standard deviation.

Serum concentrations of sFLCs at the start of the first HCO treatment did not differ significantly between patients with κ and those with λ (8.6 ± 5.0 and 10.1 ± 10.8 g/L, respectively; *p* = 0.71). The average (SD) reduction in sFLCs during the first HCO dialysis of 6 h duration was −61.4 ± 19.1% in patients with κ and −55 ± 16.7% in those with λ (*p* = 0.78) (Figure 1).

### 3.3. Clearance of sFLCs by HCO Dialysis

**Figure 2:**
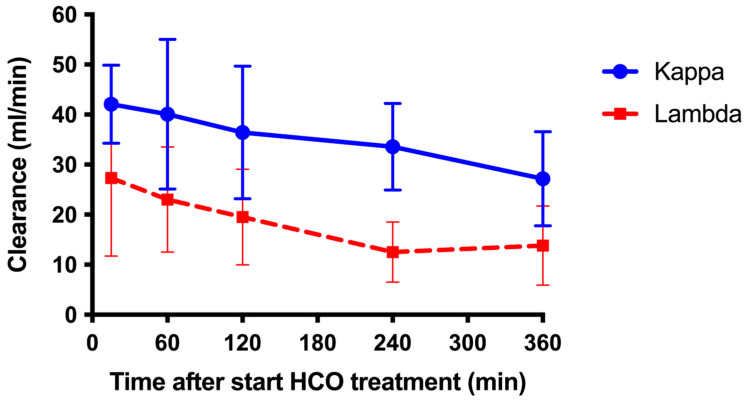
Clearance of serum FLCs. Average clearance of sFLCs during the first 2 HCO treatments for kappa (10 patients) and lambda (5 patients). Error bars indicate standard deviation.

For this analysis, clearances of the first and second HCO treatment were averaged for each individual patient. The clearance of κ was significantly higher compared to the clearance of λ at almost all time points (Figure 2). At 15 min after the start of HCO dialysis, the average clearance of sFLCs was 42.1 ± 8.5 and 27.4 ± 15.6 mL/min for κ and λ, respectively (*p* < 0.01). During HCO dialysis, the clearance of both κ and λ gradually decreased to 27.2 ± 11.3 for κ and 13.8 ± 7.9 mL/min for λ after 6 h of HCO treatment (*p* = 0.042 for the difference between κ and λ).

### 3.4. Adverse Effects

**Figure 3:**
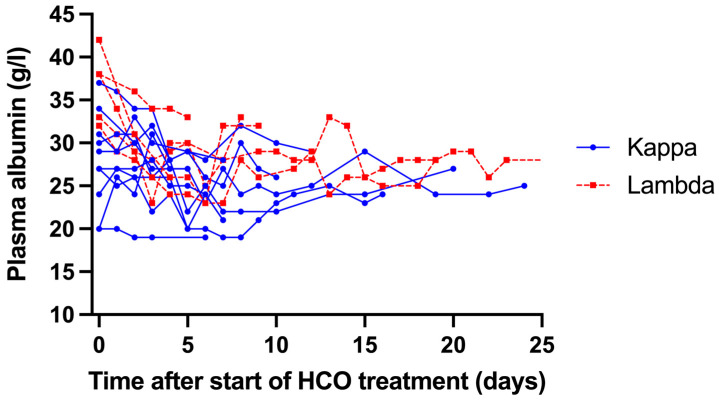
Course of plasma albumin levels during the complete HCO treatment period. Each line represents an individual patient.

All patients tolerated the HCO treatment well. Hemodynamic instability and clotting of the HCO filter necessitating premature termination of HCO treatment were not observed. Plasma albumin concentrations fell from 31.2 ± 6.5 to 26.1 ± 4.0 g/L (*p* = 0.0003) during the first 7 days after the start of HCO treatment and stabilized thereafter (Figure 3). None of the patients received albumin substitution during or in-between HCO dialysis sessions.

### 3.5. Long-Term Outcomes

In 11 of the 15 patients (73%), renal function recovered to such an extent that hemodialysis could be stopped (Table 1). Two of these patients died in the first year after the start of HCO treatment due to progression of multiple myeloma despite anti-myeloma treatment (patient 6 and patient 15 in Table 1). At the time of death, the eGFR in these patients was 74 and 18 mL/min/1.73 m^2^, respectively. Two of the four patients whose renal function did not recover died in the first year after the start of HCO treatment (patient 1 and patient 8 in Table 1). 

There were no significant differences between patients with and those without renal recovery with regard to sex (18% and 25% female, respectively), age (mean 68.7 ± 9.8 and 68.3 ± 12.8 years, respectively) and sFLC type (36% and 25% λ, respectively). The sFLC concentration at diagnosis was non-significantly lower in patients whose renal function subsequently recovered compared to those without renal recovery (median 5.7 [IQR 2.5–12.9] and 14.7 [IQR 10.7–16.1], respectively; *p* = 0.15). As expected, the number of HCO treatments (median 9.3 [IQR 5–14] and 13 [IQR 8–33], respectively; *p* = 0.07) and duration of HCO treatment (median 10 [IQR 6–20] and 20 [IQR 12–63] days, respectively; *p* = 0.08) was shorter in patients with renal recovery compared to those without renal recovery.

**Figure 4:**
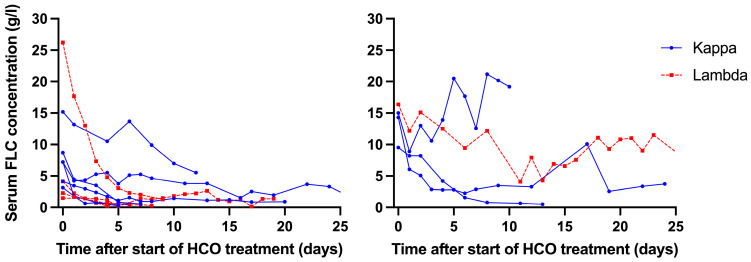
Course of sFLC levels during the complete HCO treatment period for patients in whom renal function recovered (left panel) and in those in whom renal function did not recover (right panel). Each line represents an individual patient.

As shown in Figure 4, most patients whose renal function recovered had a relatively fast decrease in sFLCs, with none of these patients having sFLC levels > 5 g/L beyond 14 days after the start of HCO treatment. In contrast, three out of the four patients whose renal function did not recover had sFLC levels > 5 g/L at any time beyond 2 weeks after the start of HCO treatment. In these three patients, the relative reduction in sFLCs during the first HCO treatment did not differ significantly from patients whose renal function recovered (−52.7 ± 21.2% versus −63.3 ± 17.9%; *p* = 0.43). In other words, the absence of a substantial decrease in sFLCs over time in these three patients could not be explained by less efficient HCO treatment.

## 4. Discussion

Rapid reduction in sFLCs is essential for the recovery of renal function in patients with acute renal failure due to myeloma cast nephropathy [1,2,3]. This study shows that a single 6 h hemodialysis session with an HCO dialyzer effectively lowers plasma concentrations of both κ and λ. It also shows that the clearance of κ appears to be greater than that of λ and that clearances of both κ and λ gradually decrease during the treatment. Finally, our study suggests that patients whose renal function did not recover had less of a reduction in sFLC levels during HCO treatment. This could not be explained by a less effective removal of sFLCs by HCO treatment and is probably due to a suboptimal hematological response to anti-plasma cell therapy. This study confirms the results of previous studies showing that HCO hemodialysis effectively reduces sFLCs [3,4,6,7,8,9,10,11,12,13,14]. In our study, the average reductions in FLCs (−61.4% for κ; −55% for λ) during a 6 h treatment were slightly lower than those reported by Hutchinson et al. (−7% for κ; −72% for λ) during an 8 h session [4] and those reported by Bridoux et al. during a 5 h treatment (−77% for κ; −63% for λ) [12]. Notably, Hutchinson et al. used two HCO dialyzers, each with a surface area of 1.1 m^2^, in series, whereas we used a single HCO dialyzer with a surface area of 2.1 m^2^. Interestingly, we found that the clearance of κ was significantly higher than the clearance of λ, probably thanks to the lower molecular size of κ (22.5 Da) compared to λ (45 Da), since molecular weight is a major determinant of diffusion. Despite the lower clearance of λ, the reduction in serum levels was similar for κ and λ. We speculate that the discrepancy between clearance and the course of serum levels is explained by a greater adherence of λ to the HCO membrane compared to λ. Adherence of sFLCs to the HCO membrane with clogging of pores of the semipermeable membrane may also explain the decrease in clearance during HCO treatment that was observed for both κ and λ. 

Hemodialysis with HCO membranes is associated with significant intradialytic loss of albumin [8,14]. In two randomized controlled studies, albumin was administered intravenously in a large proportion of patients to avoid hypoalbuminemia [4,12]. In the present study, we did not administer albumin and, indeed, observed a significant reduction in plasma albumin concentration in the first week of HCO treatment. In most patients, albumin levels increased in the second week of HCO treatment and, for those that were still treated with HCO dialysis beyond 2 weeks, stabilized thereafter. We did not observe clinical problems (e.g., intradialytic hemodynamic instability) due to hypoalbuminemia and, therefore, do not recommend the routine use of albumin infusion. 

Neither of the two randomized controlled studies comparing HCO hemodialysis with conventional hemodialysis using high-flux dialyzers showed a statistically significant difference for the primary endpoint of independence of dialysis at 3 months between the two arms [4,12]. However, Bridoux et al. observed significantly higher hemodialysis independence rates in the HCO hemodialysis group at 6 and 12 months [12]. Although our observational study was too small to draw firm conclusions on renal recovery, our study suggests that patients whose renal function did not recover showed less of a reduction in sFLC levels during HCO treatment. Three out of the four patients whose renal function did not recover consistently had pre-HCO treatment sFLC concentrations of >5 g/L. This was most probably due to treatment resistance of multiple myeloma. In our opinion, it is not realistic to expect that renal function will recover with HCO treatment if pre-dialysis sFLC concentrations remain high despite anti-plasma cell therapy treatment. We suggest that the lack of recovery of renal function in patients with persistently high sFLC concentration should primarily be considered a suboptimal hematological response to anti-plasma cell therapy rather than a lack of efficacy of HCO treatment. In this regard, early assessment of the hematological response based on serial sFLC measurements is crucial [1]. We agree with Bridoux and co-authors that, if a rapid and deep hematological response is not achieved, reinforcing the anti-plasma cell therapy should be considered [1]. In this regard, the anti-CD38 monoclonal antibody daratumumab is promising in rapidly reducing sFLCs [15]. It has been argued that the fast inhibition of the production of sFLCs by anti-CD38 antibodies could push HCO treatment to the side [1]. Alternatively, one could argue that the rapid inhibition of FLC production combined with early effective removal of circulating sFLCs by HCO treatment will reduce the sFCL burden to the kidney and, thus, may further improve outcome.

Limitations of this study include the relatively small number of patients, the lack of renal biopsies and the absence of experimental validation, e.g., by membrane analysis, of our hypothesis that λ sFLCs have a greater adherence to the HCO membrane. Additionally, the single-arm observational design limits the ability to generalize results and draw causal inferences. Therefore, prospective randomized studies with a larger number of patients are needed for a final conclusion. Strengths include the detailed characterization of the course of sFLCs during the complete course of HCO treatment, including measurement of the clearance of sFLCs. Importantly, the interval between diagnosis and start of HCO treatment in our study was relatively low (4.4 days for newly diagnosed patients and 1.4 days for patients with a relapse). The interval between diagnosis and start of anti-plasma cell therapy was even shorter because this therapy was initiated before HCO treatment was started. In our opinion, the removal of sFLCs by HCO hemodialysis is only meaningful if, at the same time, the production of sFLCs is inhibited by highly effective anti-plasma cell therapy. 

## 5. Conclusions

The reduction in sFLCs during a single HCO treatment was similar for κ and λ, although the clearance of κ was significantly higher than of λ, probably due to the lower molecular size of κ. We speculate that this discrepancy is explained by greater adherence of λ to the HCO membrane. Patients whose renal function did not recover showed less of a reduction in sFLC levels during HCO treatment, most probably due to a suboptimal hematological response to anti-plasma cell therapy.

## Figures and Tables

**Table 1:** Patient characteristics.

ID	M/F	Age (yr)	sFLC Type	New Diagnosis (N) or Relapse (R)	Chemotherapy	sFLC at Diagnosis (g/L)	Nr of HCO Treatments	Duration of HCO Treatment (days)	sFLC Reduction During First Treatment (%)	Mean sFLC Reduction During First 2 Treatments (%)	Patient Status at 1 yr After Start of HCO Treatment	Independence from Dialysis at Stop of HCO Treatment	Renal Function at Follow-Up (eGFR in mL/min/1.72 m^2^; Creatinine Clearance in mL/min)		
													3 Months After Start of HCO Treatment	6 Months After Start of HCO Treatment	12 Months After Start of HCO Treatment
1	F	66	Kappa	F	Bortezomib, dexamethasone	9.5	7	13	33.0	58.6	Died (3 months after start HCO)	No	Dialysis- dependent	Dialysis- dependent	Dialysis- dependent
2	M	70	Lambda	F	Bortezomib, dexamethasone, doxorubicin	16.4	39	75	50.2	46.6	Alive	No	Dialysis-dependent	Dialysis-dependent	Dialysis- dependent
3	M	63	Kappa	F	Bortezomib, dexamethasone, doxorubicin	8.7	5	7	73.0	76.9	Alive	Yes	eGFR 29 CC 32	eGFR 42 CC 55	eGFR 37 CC 45
4	M	53	Lambda	R	Bortezomib, dexamethasone	2.3	4	6	59.4	62.6	Alive	Yes	eGFR 51 CC 68	eGFR 56 CC	NA Died
5	M	62	Kappa	R	Bortezomib, dexamethasone	1.5	6	6	64.8	61.1	Alive	Yes	eGFR 56 CC 16	eGFR NA CC NA	eGFR: NA CC NA
6	F	70	Lambda	F	Bortezomib, dexamethasone	26.2	10	10	29.0	29.3	Died (5 months after start of HCO)	Yes	eGFR 98 CC 23	NA Died	NA Died
7	M	81	Kappa	R	Bortezomib, prednisolone, melphalan	4.2	12	20	74.2	70.7	Alive	Yes	eGFR 25 CC 30	eGFR NA CC NA	NA
8	M	53	Kappa	R	Bortezomib, dexamethasone, lenalidomide	15.0	12	12	43.4	37.5	Died (1 month after start of HCO)	No	NA Died	NA Died	NA Died
9	M	64	Kappa	F	Bortezomib, dexamethasone	15.2	9	12	46.6	53.0	Alive	Yes	eGFR 22 CC NA	eGFR 29 CC NA	eGFR 35 CC NA
10	M	79	Kappa	F	Bortezomib, dexamethasone	7.2	5	5	85.4	66.1	Alive	Yes	eGFR 9 CC 19	eGFR 11 CC 15	eGFR 12 CC 17
11	M	62	Kappa	F	Bortezomib, dexamethasone	7.2	16	36	81.8	63.5	Alive	Yes	eGFR 46 CC 65	eGFR 51 CC NA	eGFR 57 CC 72
12	M	84	Kappa	F	Bortezomib, dexamethasone	14.3	14	25	56.7	49.8	Alive	No	Dialysis-dependent	Dialysis-dependent	Dialysis- dependent
13	M	63	Kappa	F	Bortezomib, dexamethasone, thalidomide	3.1	5	6	63.7	56.3	Alive	Yes	eGFR 31 CC 40	eGFR 36 CC 86	eGFR 32 CC 64
14	F	64	Lambda	R	Bortezomib, dexamethasone, daratumumab	4.1	9	9	71.4	68.5	Alive	Yes	eGFR 23 CC 27	eGFR 29 CC 26	eGFR 27 CC 39
15	M	79	Lambda	F	Bortezomib, dexamethasone, daratumumab	1.5	17	19	66.3	59.1	Died (1 month after start HCO)	Yes	NA Died	NA	NA

Abbreviations: CC: creatinine clearance; eGFR: estimated glomerular filtration rate; ID: patient identification number; HCO: high cut-off; NA: not available; sFLC: serum free light chain.

## Data Availability

Data related to this study are available upon reasonable request to the corresponding author.

## References

[1] Bridoux F., Leung N., Belmouaz M., Royal V., Ronco P., Nasr S.H., Fermand J.P. (2021). Management of acute kidney injury in symptomatic multiple myeloma. Kidney Int..

[2] Leung N., Rajkumar S.V. (2023). Multiple myeloma with acute light chain cast nephropathy. Blood Cancer J..

[3] Hutchison C.A., Bradwell A.R., Cook M., Basnayake K., Basu S., Harding S., Hattersley J., Evans N.D., Chappel M.J., Sampson P. (2009). Treatment of acute renal failure secondary to multiple myeloma with chemotherapy and extended high cut-off hemodialysis. Clin. J. Am. Soc. Nephrol..

[4] Hutchison C.A., Cockwell P., Moroz V., Bradwell A.R., Fifer L., Gillmore J.D., Jesky M.D., Storr M., Wessels J., Winearls C.G. (2019). High cutoff versus high-flux haemodialysis for myeloma cast nephropathy in patients receiving bortezomib-based chemotherapy (EuLITE) a phase 2 randomised controlled trial. Lancet Haematol..

[5] Kleber M., Ihorst G., Terhorst M., Koch B., Deschler B., Wäsch R., Engelhardt M. (2011). Comorbidity as a prognostic variable in multiple myeloma: Comparative evaluation of common comorbidity scores and use of a novel MM–comorbidity score. Blood Cancer J..

[6] Hutchison C.A., Cockwell P., Stringer S., Bradwell A., Cook M., Gertz M.A., Dispenzieri A., Winters J.L., Kumar S., Rajkumar S.V. (2011). Early reduction in serum-free light chains associates with renal recovery in myeloma kidney. Clin. J. Am. Soc. Nephrol..

[7] Hutchison C.A., Cockwell P., Reid S., Chandler K., Mead G.P., Harrison J., Hattersley J., Evans N.D., Chappell M.J., Cook M. (2007). Efficient removal of immunoglobulin free light chains by hemodialysis for multiple myeloma: In vitro and In vivo studies. J. Am. Soc. Nephrol..

[8] Hutchison C.A., Harding S., Mead G., Goehl H., Storr M., Bradwell A., Cockwell P. (2008). Serum free-light chain removal by high cutoff hemodialysis: Optimizing removal and supportive care. Artif. Organs.

[9] Hutchison C.A., Heyne N., Airia P., Schindler R., Zickler D., Cook M., Cockwell P., Grima D. (2012). Immunoglobulin free light chain levels and recovery from myeloma kidney on treatment with chemotherapy and high cut-off haemodialysis. Nephrol. Dial. Transplant..

[10] Peters N.O., Laurain E., Cridlig J., Hulin C., Cao-Huu T., Frimat L. (2011). Impact of free light chain hemodialysis in myeloma cast nephropathy: A case-control study. Hemodial. Int..

[11] Zannetti B.A., Zamagni E., Santostefano M., De Sanctis L.B., Tacchetti P., Mancini E., Pantani L., Brioli A., Rizzo R., Mancuso K. (2015). Bortezomib-based therapy combined with high cut-off hemodialysis is highly effective in newly diagnosed multiple myeloma patients with severe renal impairment. Am. J. Hematol..

[12] Bridoux F., Carron P.-L., Pegourie B., Alamartine E., Augeul-Meunier K., Karras A., Joly B., Peraldi M.-N., Arnulf B., Vigneau C. (2017). Effect of high-cutoff hemodialysis vs. conventional hemodialysis on hemodialysis independence among patients with myeloma cast nephropathy. JAMA.

[13] Steiner N., Hamid A.A., Kronbichler A., Neuwirt H., Myslivecek M., Kollar M., Lachmanova J., Rysava R., Hruskova Z., Spicka I. (2021). Real world analysis of high-Cut-Off (Hco) hemodialysis with bortezomib based backbone therapy in patients with multiple myeloma and acute kidney injury. J. Nephrol..

[14] van Gelder M.K., Abrahams A.C., Joles J.A., Kaysen G.A., Gerritsen K.G.F. (2018). Albumin handling in different hemodialysis modalities. Nephrol. Dial. Transplant..

[15] Hughes M.S., Balev M., Radhakrishnan J., Bhutani D., Mapara M., Lentzsch S., Chakraborty R. (2025). Improved outcomes of myeloma cast nephropathy in newly diagnosed multiple myeloma with modern anti-myeloma therapies. Eur. J. Hematol..

